# Tax on Lawyers, Physicians and Dentists

**Published:** 1853-10

**Authors:** 


*Tax on Lawyers, Physicians and Dentists.*—Judge Lomax has pronounced the tax on lawyers, physicians and dentists, in Virginia, to be unconstitutional. In consequence of which, the town council of Fredericksburg have directed, that such taxes for 1852 be refunded. A similar law was passed by the Legislature of Maryland, a few years since, but, we believe, was never carried into effect, and has subsequently been repealed.

